# P-34. No Significant Interaction Between Serum Albumin Level and Carbapenem Type on 30-Day Mortality in ESBL-Producing Bacteremia: A Multicenter Cohort Study

**DOI:** 10.1093/ofid/ofaf695.263

**Published:** 2026-01-11

**Authors:** Younghee Jung, Eun-ju Jung, Jin Ju Park, Hong Sang Oh

**Affiliations:** Chungnam National University Hospital, Daejeon, Taejon-jikhalsi, Republic of Korea; Dongtan Sacred Heart Hospital, Hwaseong, Kyonggi-do, Republic of Korea; Hallym University Gangnam Sacred Heart Hospital, Seoul, Seoul-t'ukpyolsi, Republic of Korea; Hallym University Sacred Heart Hospital, Anyang-si, Kyonggi-do, Republic of Korea

## Abstract

**Background:**

The Infectious Diseases Society of America guidelines recommend using group 2 carbapenems over ertapenem in critically ill or hypoalbuminemic patients for the treatment of extended-spectrum β-lactamase (ESBL)-producing Enterobacterales. However, clinical evidence supporting the cautious use of ertapenem in hypoalbuminemic patients remains limited. We aimed to assess whether the impact of carbapenem type on 30-day mortality differed by serum albumin level in patients with ESBL-producing bacteremia.

**Figure:**
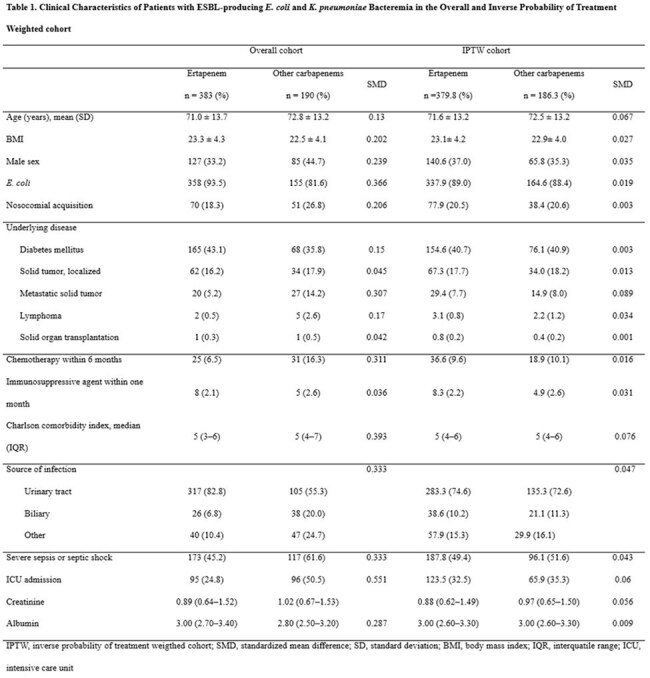
Clinical Characteristics of Patients with ESBL-producing E. coli and K. pneumoniae Bacteremia in the Overall and Inverse Probability of Treatment Weighted cohort IPTW, inverse probability of treatment weigthed cohort; SMD, standardized mean difference; SD, standard deviation; BMI, body mass index; IQR, interquatile range; ICU, intensive care unit

**Table 2.**
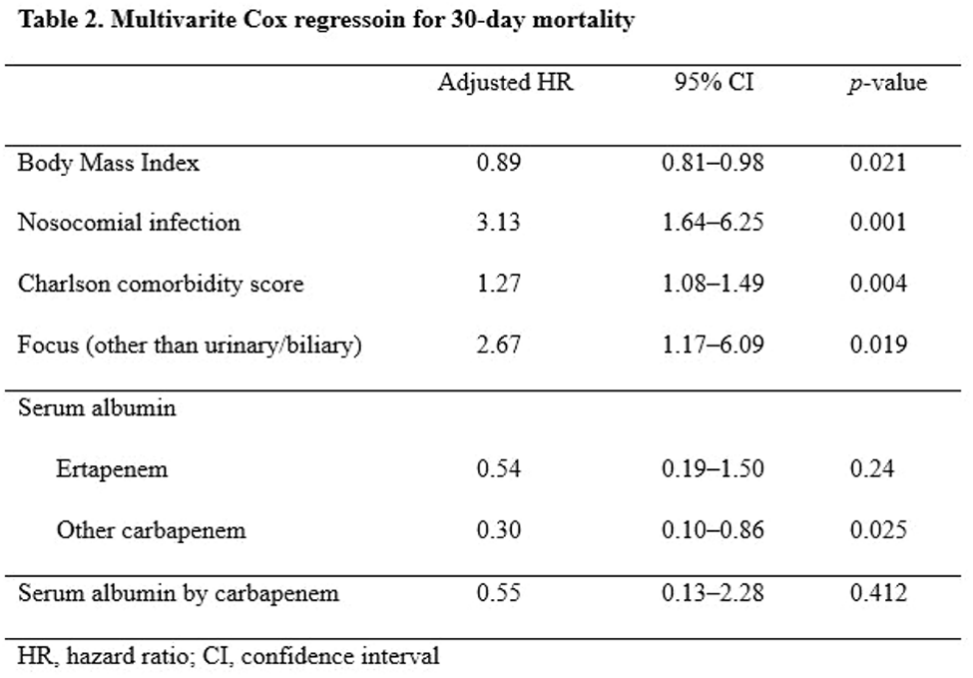
Multivarite Cox regressoin for 30-day Mortality HR, hazard ratio; CI, confidence interval

**Methods:**

We conducted a multicenter, retrospective cohort study at three university-affiliated hospitals, enrolling patients treated exclusively with either ertapenem or group 2 carbapenem due to ESBL-producing E. coli or K. pneumoniae bacteremia between 2013 and 2020. Baseline characteristics were balanced using inverse probability of treatment weighting (IPTW). Risk factors for 30-day mortality were identified with multivariate Cox regression. To explore potential effect modification, an interaction term between carbapenem type and serum albumin level was included in the model.

**Figure:**
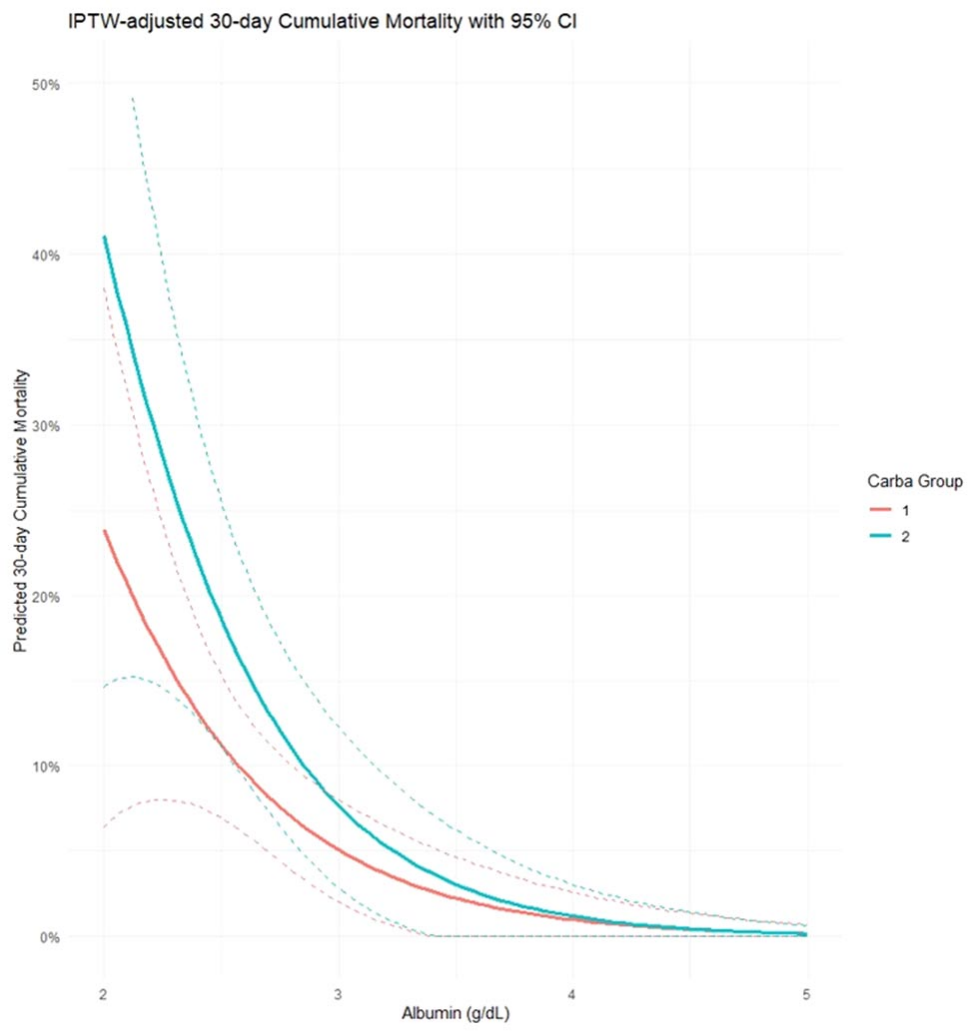
IPTW-adjusted Predicted 30-day Mortality by Albumin IPTW, Inverse probability of treatment weighted

**Results:**

A total of 566.2 weighted patients were analyzed (379.8 in ertapenem and 186.3 in group 2 carbapenems). After IPTW adjustment, baseline characteristics between the groups were well balanced. The overall 30-day mortality rate was 8.5% (47.9 deaths). Risk factors for 30-day mortality were lower body mass index (adjusted hazard ratio [aHR] 1.12, 95% confidence interval [CI] 1.02–1.23, p = 0.021), nosocomial infection (aHR 3.13, 95% CI 1.64–6.25, p = 0.001), higher Charlson comorbidity index score (aHR 1.27, 95% CI 1.10–1.49, p = 0.004), and infection source other than urinary or biliary tract (aHR 2.67, 95% CI 1.17–6.10, p = 0.019). Serum albumin was associated with 30-day mortality in group 2 carbapenem (aHR 0.34, 95% CI 0.13–0.87, p = 0.025), whereas such association was not observed in ertapenem (aHR 0.63, 95% CI 0.29–1.41, p = 0.24) in subgroup analyses. No significant interaction between carbapenem type and serum albumin level was found (p = 0.41).

**Conclusion:**

Given the absence of a significant interaction between serum albumin level and carbapenem type, ertapenem can be used regardless of serum albumin levels in patients with ESBL-producing bacteremia.

**Disclosures:**

All Authors: No reported disclosures

